# Health status instruments for patients with COPD in pulmonary rehabilitation: defining a minimal clinically important difference

**DOI:** 10.1038/npjpcrm.2016.41

**Published:** 2016-09-01

**Authors:** Harma Alma, Corina de Jong, Danijel Jelusic, Michael Wittmann, Michael Schuler, Bertine Flokstra-de Blok, Janwillem Kocks, Konrad Schultz, Thys van der Molen

**Affiliations:** 1 Department of General Practice, GRIAC Research Institute, University of Groningen, University Medical Center Groningen, Groningen, The Netherlands; 2 Klinik Bad Reichenhall, Center for Rehabilitation, Pulmonology and Orthopedics, Bad Reichenhall, Germany; 3 Department of Psychotherapy and Medical Psychology, Rehabilitation Sciences Section, University of Wuerzburg, Wuerzburg, Germany

## Abstract

The minimal clinically important difference (MCID) defines to what extent change on a health status instrument is clinically relevant, which aids scientists and physicians in measuring therapy effects. This is the first study that aimed to establish the MCID of the Clinical chronic obstructive pulmonary disease (COPD) Questionnaire (CCQ), the COPD Assessment Test (CAT) and the St George’s Respiratory Questionnaire (SGRQ) in the same pulmonary rehabilitation population using multiple approaches. In total, 451 COPD patients participated in a 3-week Pulmonary Rehabilitation (PR) programme (58 years, 65% male, 43 pack-years, GOLD stage II/III/IV 50/39/11%). Techniques used to assess the MCID were anchor-based approaches, including patient-referencing, criterion-referencing and questionnaire-referencing, and the distribution-based methods standard error of measurement (SEM), 1.96SEM and half standard deviation (0.5s.d.). Patient- and criterion-referencing led to MCID estimates of 0.56 and 0.62 (CCQ); 3.12 and 2.96 (CAT); and 8.40 and 9.28 (SGRQ). Questionnaire-referencing suggested MCID ranges of 0.28–0.61 (CCQ), 1.46–3.08 (CAT) and 6.86–9.47 (SGRQ). The SEM, 1.96SEM and 0.5s.d. were 0.29, 0.56 and 0.46 (CCQ); 3.28, 6.43 and 2.80 (CAT); 5.20, 10.19 and 6.06 (SGRQ). Pooled estimates were 0.52 (CCQ), 3.29 (CAT) and 7.91 (SGRQ) for improvement. MCID estimates differed depending on the method used. Pooled estimates suggest clinically relevant improvements needing to exceed 0.40 on the CCQ, 3.00 on the CAT and 7.00 on the SGRQ for moderate to very severe COPD patients. The MCIDs of the CAT and SGRQ in the literature might be too low, leading to overestimation of treatment effects for patients with COPD.

## Introduction

Chronic obstructive pulmonary disease (COPD) is the third leading cause of death.^1^ Spirometry is required to make the diagnosis.^2^ Its parameter forced expiratory volume in 1 s (FEV_1_) has, however, a weak correlation with symptoms and disease impact, which are factors captured by health status instruments.^3^ Health status has become an important goal in the management of COPD.^2^ Multiple instruments exist that measure health status with the Clinical COPD Questionnaire (CCQ), COPD Assessment Test (CAT) and St. George’s Respiratory Questionnaire (SGRQ) most frequently used.^4–6^ These tools are important in assessing treatment effectiveness. Therefore, clinically relevant change as an outcome of the questionnaires has become pivotal. The minimal clinically important difference (MCID) is a parameter that assesses clinically relevant change. It is defined as ‘the smallest difference in score, which patients perceive as beneficial and which would mandate a change in the patient’s management’.^7^

Multiple methods for determining the MCID exist, clustered into anchor-based and distribution-based approaches.^8–10^ Anchor-based approaches require change in health status to be compared with another measure of clinical change, such as a global rating of change (GRC) assessment (patient-referencing); the appearance of health events in the time of change (criterion-referencing); and/or a related instrument with known MCID (questionnaire-referencing).^8^ Distribution-based methods require comparison of change with a statistical measure of variability of this change, such as the standard error of measurement (SEM) or half the s.d. (0.5s.d.).^8,11,12^ Anchor-based methods are preferred, as they convey clinical significance, yet distribution-based approaches are quicker to use.^9,10^ A golden standard has not been defined.

Different methods will lead to a range of estimates.^8,10,13^ A pledge has been made for an overall body of evidence to agree upon an MCID or to use multiple in practice.^8,10,13,14^ This body of evidence should consist of relevant patient-reported anchors and clinical trial data.^10,15^ However, selecting appropriate anchors is problematic, since this commonly used method is highly dependent on the correlation between instruments (preferably ⩾0.50) as well as the accuracy of the anchor instrument’s MCID.^10,15^

Existing evidence for the MCID of the CCQ suggests a value of 0.40, which is equivalent to 7% of the scale (range: 0–6).^16–19^ The anchor-based methods patient-referencing, criterion-referencing and questionnaire-referencing, with the SGRQ, CAT and chronic respiratory questionnaire (CRQ) as anchors, were separately applied in either a Dutch prednisolone trial following acute exacerbation, in pulmonary rehabilitation (PR) for both COPD and non-COPD patients or in a Greek primary and secondary care population.^16–18^ The SEM and 0.5s.d. techniques were applied too.^17–19^ None of the studies combined all of the approaches in the same COPD population. The MCID for the domain scores on the CCQ has not been established either.

The MCID of the CAT was summarised into two points, equivalent to 5% of the scale (range: 0–40).^5,19–21^ Both anchor-based and distribution-based techniques were applied in a PR setting, for acute exacerbation COPD patients and for outpatients. Criterion-referencing has not been specifically applied for the CAT, nor have all methods been applied simultaneously.

The MCID for the SGRQ is set at four points, which is 4% of the scale (range: 0–100).^6,22,23^ Expert-based ratings, patient-referencing, criterion-referencing and the use of the 6-min walking distance (6MWD) and the CRQ as anchors have been applied in various studies on asthma and COPD patients. These studies are from many years ago; therefore, a recent study on severe COPD patients, who underwent bronchoscopic lung volume reduction, claimed the MCID of the SGRQ to be >7 points.^24^ It used FEV_1_, 6MWD and residual volume as anchors combined with distribution-based methods. Estimates on the MCID of the SGRQ seem inconsistent. None of the methods have been applied at once, nor is the MCID of domain scores of the SGRQ investigated.

The MCIDs of health status tools are necessary for physicians and researchers to evaluate therapy results and clinical trials. Expanding the body of evidence for the MCID remains of major importance. This study is the first study to investigate the MCID of the CCQ, CAT and SGRQ simultaneously in pulmonary rehabilitation (PR) using the largest array of methods. It examines the impact of using anchors and distribution-based methods to determine an instrument’s MCID. The domain scores on the CCQ and SGRQ are investigated for their MCID as well, which is a new development.

## Results

### Patient characteristics and health status

In total, 611 patients participated in the RIMTCORE trial, among whom 50 discontinued the study.^25^ Out of the remaining participants, 451 met the inclusion criteria for the current MCID study. Mean age was 57.87±6.56 years, 65% were male and 50/39/11% were GOLD II/III/IV (Table 1). CCQ, CAT and SGRQ were normally distributed at T0 and T1; change scores were normally distributed for CCQ, CAT and SGRQ symptom scores. Floor and ceiling effects were negligible, except for the CCQ mental domain. There were no missing health status questionnaires at T0. There were four missing participants for the SGRQ, 1 for the CCQ and 2 for the CAT at T1. Pair-wise deletion was applied. Mean baseline scores were 2.86±1.17 (CCQ), 20.23±7.33 (CAT) and 50.69±17.33 (SGRQ), with significant improvement after PR of, respectively, 0.58 (95% confidence interval (CI) 0.50–0.67), 3.11 (95% CI 2.59–3.63) and 9.04 (95% CI 7.92–10.17; Table 2).

### MCID: patient-referencing

A GRC score was missing for one patient. Correlations between GRC and health status instruments were significant, with *r*=0.29 (CCQ), 0.23 (CAT) and 0.30 (SGRQ). In total, 12 patients showed deterioration on the GRC (GRC⩽−2). No, or hardly any, improvement (GRC=0, or +1) was experienced by 21.7% (*n*=98). Minimal improvement (GRC=+2 and +3) was seen in 43.5% of patients (*n*=196), whereas moderate (GRC=+4 and +5) and major improvements (GRC=+6 and +7) represented, respectively, 27.3% (*n*=123) and 5.5% (*n*=25; Table 3). At the threshold for minimal clinically relevant improvement (GRC=+2 or +3), mean CCQ, CAT and SGRQ change scores were, respectively, 0.56 (95% CI 0.44–0.68), 3.12 (95% CI 2.37–3.86) and 8.40 (95% CI 6.73–10.07). Mean improvements for these patients on the CCQ domains were 0.55 (symptoms), 0.55 (functional) and 0.58 (mental), and for the SGRQ domains they were 13.12 (symptoms), 5.98 (activity) and 8.24 (impact).

### MCID: criterion-referencing

During PR, 10% of patients (*n*=45) experienced an exacerbation. There were no missing data. Mean differences between both groups at baseline were 0.62 (95% CI 0.27–0.98) for CCQ, 2.96 (95% CI 0.71–5.20) for CAT and 9.28 (95% CI 3.99–14.56) for SGRQ, with significantly higher scores for patients with an exacerbation. Significant domain differences were 0.47, 0.67 and 0.86 for the respective CCQ symptoms, functional and mental domains; and 10.61 and 9.93 for the SGRQ activity and impact domains.

### MCID: questionnaire-referencing

Significant correlations between total change scores were 0.63 (SGRQ versus CCQ), 0.54 (SGRQ versus CAT) and 0.59 (CCQ versus CAT; Supplementary Table 1, online supplement). Using the original anchor estimates from the literature (CCQ=0.40, CAT=2.00 and SGRQ=4.00), the various questionnaire-referencing results including 95% CI resulted in the following ranges: 0.42–0.53 (CAT as anchor) and 0.28–0.50 (SGRQ as anchor) for CCQ; 2.14–3.00 (CCQ as anchor) and 1.46–3.00 (SGRQ as anchor) for CAT; and 6.86–8.30 (CCQ as anchor) and 6.98–8.48 (CAT as anchor) for SGRQ (Table 4, see also Supplementary figures 1-6 of the online supplement). Using averaged estimates from the other MCID approaches in this study (CCQ=0.50, CAT=3.00 and SGRQ=7.00), the results including 95% CI are 0.53–0.61 (CAT as anchor) and 0.44–0.60 (SGRQ as anchor) for CCQ; 2.54–3.08 (CCQ as anchor) and 2.32–3.00 (SGRQ as anchor) for CAT; and 7.79–8.90 (CCQ as anchor) and 8.00–9.47 (CAT as anchor) for SGRQ (Table 4, see also Supplementary figures 1-6 of the online supplement).

### MCID: distribution-based approach

The SEM for the CCQ, CAT and SGRQ was 0.29, 3.28 and 5.20, respectively; the 1.96SEM was 0.56, 6.43 and 10.19, respectively; and the 0.5s.d. was 0.46, 2.80 and 6.06, respectively.

### Pooled MCID estimates

**Figure 1:**
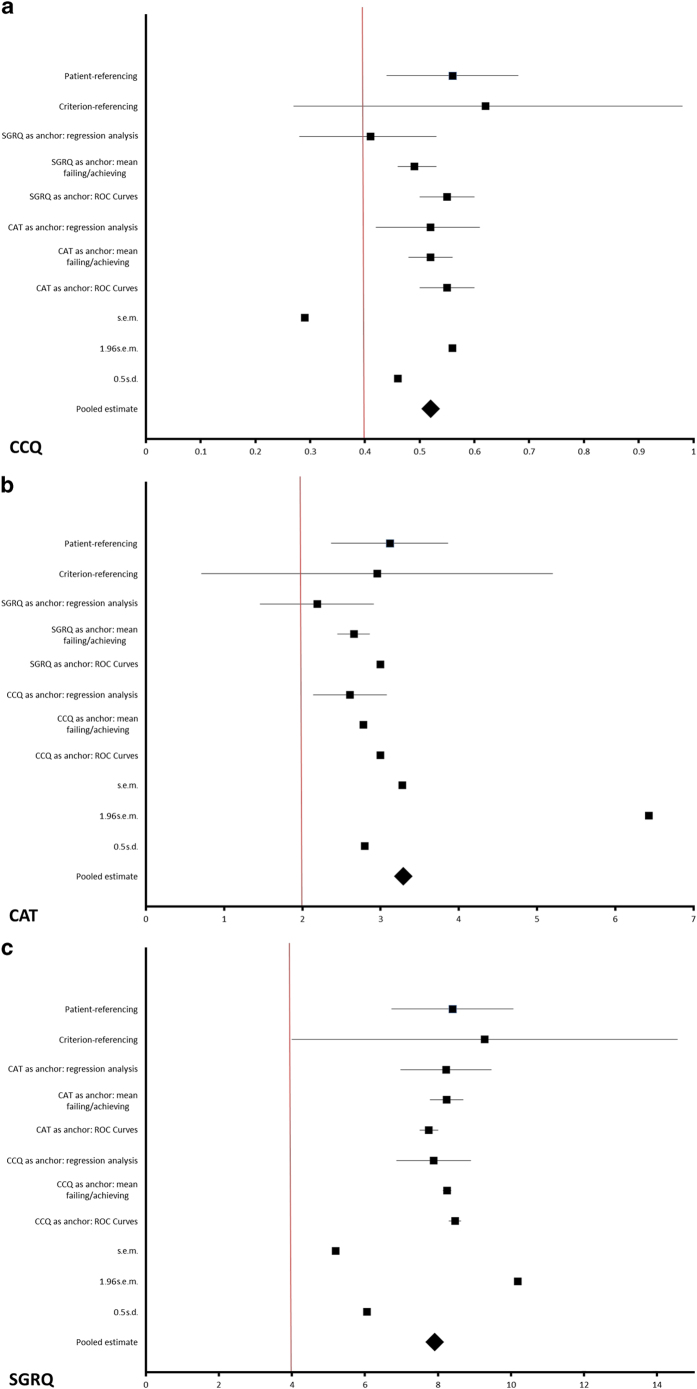
(**a**–**c**) Summary plots of the CCQ, CAT and SGRQ MCID estimates. The horizontal plots represent the MCID estimates derived in this study, classified per method. Where appropriate the estimates include the 95% confidence interval. The red vertical line resembles the MCID estimate as obtained from the literature.

The weighted MCID estimates were 0.52 (CCQ), 3.29 (CAT) and 7.91 (SGRQ). Results for the domains were 0.51 (symptoms), 0.61 (functional status) and 0.72 (mental) for CCQ, and 13.12 (symptoms), 8.30 (activities) and 9.09 (impact) for SGRQ. Results from all approaches are visualised in Figure 1a–c (see also online Supplementary Table 2).

### Power analysis

*Post hoc* analysis demonstrates the power of the study to be over 90% based upon the number of cases (*N*=451), *α* 0.05 and effect sizes for CCQ (0.50), CAT (0.43) and SGRQ (0.53).

## Discussion

### Main findings

The use of anchor- and distribution-based methods in this study resulted in MCID ranges of 0.28–0.62 for CCQ, 1.46–6.43 for CAT and 5.20–10.19 for SGRQ. The pooled MCID estimates derived from the various methods in this study (0.52 for CCQ, 3.29 for CAT and 7.91 for SGRQ) are similar or slightly higher compared with the literature. In general, results from the patient-referencing method were somewhat comparable to criterion- and questionnaire-referencing. The adjusted MCID cut-off points of the anchors (SGRQ=7.00, CCQ=0.50 and CAT=3.00) in the questionnaire-referencing approach had slightly better correspondence with patient- and criterion-referencing. The distribution-based method 0.5s.d. was best comparable to anchor-based results. The SEM was inconsistent, and the 1.96SEM was much more conservative for CAT and SGRQ, although not for CCQ.

### Interpretation of findings in relation to previously published work

The pooled MCID estimate for the CCQ in our study (0.52) is slightly higher than the literature estimate. Patient- and criterion-referencing in our study used comparable methodology,^16,18^ but resulted in more conservative estimates. Differences in participants’ age, baseline CCQ score and period of measurement possibly influenced this. Our study included younger patients with more severe baseline scores. Furthermore, exacerbations might not be a minor event for the included patients. Questionnaire-referencing resulted in ranges of 0.28–0.60 (SGRQ anchor) and 0.42–0.61 (CAT anchor), which to some extent matched the results of Kon *et al*.^17^ for COPD, as well as of Canavan et al. for other respiratory diseases.^18^ With regard to the distribution-based methods, the 1.96SEM and 0.5s.d. were best comparable to results from anchor-based approaches, but slightly higher than the results of Kocks *et al*.^16^ The 0.5s.d. matched earlier results.^17,18^ Domain MCID scores are approximately equivalent to MCID estimates of the total CCQ score, although the mental domain was higher possibly because of floor and ceiling effects in the current study.

Almost all MCID estimates including the pooled estimate (3.29) for CAT were higher than the suggested two points in the literature. Patient-referencing resulted in a much higher estimate compared with Dodd *et al*.^21^ and Kon *et al*.^20^, who used both a GRC with just five anchor questions. Preferably, more answering options on the anchor question should be used to provide the full spectrum.^26^ Criterion-referencing in our study was comparable to other anchor-based approaches. This method has not been performed for the CAT before. Questionnaire-referencing resulted in ranges of 1.46–3.00 (SGRQ anchor) and 2.14–3.08 (CCQ anchor). The use of the original MCID of the SGRQ as anchor provided the lowest estimates for the CAT, just as for CCQ. Possibly, the MCID of the SGRQ is not as solid as claimed. The ranges found in our study matched results from Kon *et al*.,^20^ but they are higher than the two points summarised. Earlier, CAT has been mapped to the SGRQ, resulting in an MCID of 1.60.^5,21^ It seems that this derived from multiplication of the SGRQ MCID with a factor 40/100, which is rather unusual. A similar exercise for CCQ would result in an MCID of 0.24, far below current estimates. The distribution-based methods SEM and 0.5s.d. matched results from anchor-based approaches. The 1.96SEM is much higher and lacked correspondence.

All MCID estimates for SGRQ were higher than the four points from the literature.^22^ MCIDs of the domain scores on the SGRQ (except for symptoms) seemed comparable to the estimate for the total score. The suggested literature MCID of the SGRQ originates from patient-referencing in two studies, featuring the use of Salmeterol in COPD and Nedocromil Sodium in Asthma,^27,28^ in which a limited five-point GRC scale was used to review therapy effects.^23^ Osman *et al*. report the results of criterion-referencing comparing SGRQ scores between patients re-admitted within 12 months and those who were not, resulting in an MCID estimate of 4.80^29^. The results in the current study are double the original MCID estimates, which date back to the nineties. Differences in study setting, age of patients, time period of measurement and different health event criterion may have influenced this large difference. Poor methodologic quality of the patient-referencing approach might be another explanation.

Questionnaire-referencing provided ranges of 6.98–9.47 (CAT anchor) and 6.86–8.90 (CCQ anchor), which is somewhat comparable to patient- and criterion-referencing. MCID estimates from the adjusted questionnaire-referencing approach were slightly higher and better comparable to the other anchor-based approaches. Upon development of the SGRQ, a hypothesised multivariate model estimated a 6% mean difference on the 6MWD (22 m) to be equivalent to four points on the SGRQ.^6^ However, nowadays the MCID of the 6MWD is considered to be doubled^30^. Schünemann et al. found a change of 3.05 on the SGRQ to match the MCID of the CRQ dyspnoea domain;^31^ however, this is only a measure for dyspnoea and not the complete health status concept. It could have underestimated the MCID of the SGRQ severely. Recently, a study by Welling *et al*. suggested the MCID of the SGRQ to be over seven points for severe COPD patients undergoing bronchoscopic lung volume reduction using both anchor-based methods and distribution-based methods.^24^ Our current study includes moderate to very severe COPD patients in pulmonary rehabilitation. However, the results overlap one another using different anchors.

The SEM and 0.5s.d. for the SGRQ are both lower than the anchor-based results, whereas the 1.96SEM was higher and lacked correspondence too. Tsiligianni *et al.*^19^ calculated the 1.96SEM of the SGRQ to be 4.84, which is substantially lower than all estimates here. Jones argued that distribution-based methods were not applicable to SGRQ, as they lack agreement with anchor-based approaches, and determined a standard error of the estimate (1.3) and 0.5s.d. (8.4) based upon averaging data from 11 studies.^22^ However, this pooled 0.5s.d. matched with results in our study.

### Strengths and limitations of this study

This study applied for the first time multiple approaches to determine the MCID of the CCQ, CAT and SGRQ simultaneously in one strong data set from pulmonary rehabilitation. It is also the first study to include estimates of the possible MCIDs for domain scores of the CCQ and SGRQ as well. Estimates are valid for improvement and for patients with moderate to very severe COPD (GOLD II–IV). No patients with wild COPD (GOLD I) were included in this study. During PR too few patients deteriorated (*n*=12) to analyze the MCID for deterioration. MCIDs for improvement and deterioration may differ.^13^

Our current study has applied an ambiguous anchor-based method of questionnaire-referencing. However, this approach is widely used and accepted elsewhere to estimate another instrument’s MCID.^17,18,20^ The pooled thresholds for clinically relevant change of the CCQ, CAT and SGRQ in our study (CCQ=0.52, CAT=3.29 and SGRQ=7.91) seem different from values reported in the literature (CCQ=0.40, CAT=2.00 and SGRQ=4.00). This has had impact on the questionnaire-referencing results. Averaged MCID estimates from patient-referencing, criterion-referencing and distribution-based methods were therefore included in the analysis as cut-off values for the anchor’s MCID. The revised MCIDs of the anchor questionnaires had better correspondence with results from these other approaches. It highlights that careful selection of anchors should be considered.

A limitation of the current study is that the correlations between CAT, CCQ, SGRQ, and the GRC scale were below the lower limit to be appropriate as anchor (*r*⩾0.50). Other studies using a GRC seldom published correlation coefficients, making it unclear whether this problem is widespread. Another limitation of this study is that the PR period was three weeks, whereas the SGRQ has a recall period of one month and the CCQ has a recall period of one week. A study by Meguro *et al*.^32^ compared the shorter SGRQ-C without recall period with the original SGRQ with a specified 4-week recall period. No differences in scores between both questionnaires were observed. We therefore expect little influence of the recall period on our MCID results. Last, exacerbations during PR were used as criteria to estimate the MCID. The estimates from this approach are higher for CCQ and SGRQ compared with other methods. This might indicate that exacerbations were not a minor clinically relevant event for patients.

### Implications for future research, policy and practice

Our study demonstrated that the existing MCIDs of the SGRQ and possibly of the CAT are set too low in current practice. Using a low threshold could lead to overestimation of treatment effects. Patients currently considered to experience clinically relevant change as a result of treatment may in fact not experience this. On the other hand, a more conservative cut-off point may not approve therapy, although benefits for the patient do exist. Even though the current study adds to the body of evidence, the analysis is based on one patient group only, where many would be preferred. More studies are necessary to build a more complete body of evidence and understanding, preferably with the full scope of approaches and in different patient groups. These should also further investigate whether the MCID for the domain scores for the CCQ and SGRQ is comparable to the total score estimate. The quest in finding the gold standard for an MCID in health status tools must continue.

### Conclusion

The current study suggests that improvements exceeding 0.40 points on the CCQ, 3.00 points on the CAT and 7.00 points on the SGRQ should be considered clinically relevant for moderate to very severe COPD patients. The MCID for domain scores on the SGRQ and CCQ seems to be equivalent to these thresholds.

## Materials and methods

### Study subjects

This study is a secondary analysis of a subsample from the Routine Inspiratory Muscle Training within COPD Rehabilitation (RIMTCORE) real-life randomised controlled trial (#DRKS00004609) in the Klinik Bad Reichenhall, Center for Rehabilitation, Pulmonology and Orthopaedics in Germany.^25^ COPD patients with GOLD II–IV ⩾18 years, who gave informed consent, were included between February 2013 and July 2014. Exclusion criteria were lack of linguistic or cognitive abilities to fill out questionnaires; hyperkapnic respiratory failure with a PaCO2>50 mm Hg at rest or indication for intermittent noninvasive ventilation; contraindications for IMT (e.g., a history of recent lung surgery, fresh pulmonary embolism and history of recurrent spontaneous pneumothorax); and other severe co-morbid diseases that confer significantly greater morbidity than COPD (e.g., active cancer without successfully completed curative therapy). Patients participated in an intensive 3-week full-day inpatient rehabilitation program. The therapy components were tailored to the patients’ individual needs and included endurance and strength training, patient education, respiratory physiotherapy, psychological support, tobacco cessation and dietary counselling. The RIMTCORE trial was approved by the Ethik-Kommission der Bayerischen Landesärztekammer (#12107) and registered in the German Clinical Trial Register.

### Study design and data collection

For the current MCID study, completed data were analysed at pre (T0)- and post-inpatient rehabilitation (T1) from a subset of participants without other respiratory co-morbidities (e.g., bronchiectasis, asthma, history of bronchial carcinoma, sarcoidosis and tuberculosis) or alpha-1-antitrypsin deficiency. On all measurement occasions, parameters collected were the CCQ (weekly version), CAT (no recall period) and SGRQ (monthly version). The CCQ consists of 10 items divided over three subdomains. Item scores range from 0 to 6 (0—no impairment and 6—maximum impairment), with the total score derived from adding up item scores and dividing by 10.^4^ The CAT is an eight-item one-dimensional scale with item scores ranging from 0 to 5 (0—no impairment and 5—maximum impairment) and a total score of 0–40.^5^ The SGRQ has 50 items divided over three domains with a total score of 0–100 (0—no impairment, 100—maximum impairment).^6^ A GRC anchor question ranging from −7 to +7 was issued at T1, which required patients to assess their global health in relation to COPD compared with T0. Patient characteristics, post-bronchodilator spirometry, 6MWD and exacerbations in the 12 months before PR were available too.

### Determining the MCID: anchor-based approaches

#### Patient-referencing

Changes on the CCQ, CAT and SGRQ were categorised according to the GRC score. Scores of 0 and ±1 represented no or hardly any change; scores of ±2 and ±3 were considered minimal clinically relevant change; scores of ±4 and ±5 were considered moderate change; and scores of ±6 and ±7 were considered major change, as exemplified by Juniper *et al*.^33^ The MCID was established by calculating the mean health status change score of the patients with a minimal clinically relevant change on the GRC (±2 and ±3).

#### Criterion-referencing

The health event exacerbation during PR was used as an anchor, which was defined as worsening of COPD symptoms requiring at least treatment with oral corticosteroids and antibiotics. The difference in baseline score between patients experiencing an exacerbation and those without represented the MCID.

#### Questionnaire-referencing

Change in one instrument was anchored against change in the other two instruments, as performed earlier.^17,20^ Correlations between change scores were assessed, needing to be ⩾0.50 to be eligible as anchor.^10^ The MCID of the anchor from the literature was used as reference (CCQ=0.40, CAT=2.00 and SGRQ=4.00).^16,17,20,22^ First, scatter plots and regression analysis with the anchor change score as the independent variable were produced. Second, the mean was calculated for patients achieving or failing the anchor’s MCID. Last, receiver operating characteristics curves were plotted to identify the best change in health status to discriminate between those achieving the anchor’s MCID and those failing to achieve it.^8^ This process resulted in three estimates per anchor. The steps were repeated if the MCID estimates derived from patient-referencing, criterion-referencing and the distribution-based methods were different compared with the literature.

### Determining the MCID: distribution-based approaches

The SEM seeks correlation between single standard error units and established MCID estimates.^12^ It is calculated as SEM=*σ*_x_ √1−*r*_xx_, with *r*_xx_=the intra class coefficient and *σ*_x_=standard deviation baseline. Both the SEM and 1.96SEM were calculated, as there is no consensus on which represents the MCID best. The 0.5s.d. was determined as an equivalent of the MCID.^8,11^

### Data analysis

Data analysis was performed using SPSS20.0 (IBM, Chicago, IL, USA). Descriptive data were evaluated at T0 for frequencies and percentage, or mean and s.d. Health status data at T0 and T1 were evaluated with mean and s.d., and tested for significance of change with paired *t*-tests or Wilcoxon signed-rank tests depending on normality of distribution. Health status scores of CCQ, CAT and SGRQ were checked for floor- and ceiling effects, which were defined as less than 15% of the participants scoring in the bottom and top 10% of the maximum scale score.

#### Patient-referencing approach

Correlations between GRC and CCQ, CAT or SGRQ were assessed using Spearman's coefficients. Participants were classified according to the GRC score. Significance of change was calculated with paired *t*-tests or Wilcoxon signed-rank tests depending on normality.

#### Criterion-referencing approach

The difference in baseline score between patients with and without an exacerbation during PR was evaluated using unpaired t-tests or Mann–Whitney *U* tests depending on normality.

#### Questionnaire-referencing

Correlations were assessed using Pearson's or Spearman's coefficients depending on normality. First, scatter plots and regression analysis were performed with the anchor variable as the independent variable. Next, mean change scores of the CCQ, CAT and SGRQ were calculated for those achieving or failing the suggested MCID of the anchors. Receiver operating characteristics curves were plotted with the anchor’s MCID as the dichotomising variable. The optimal value was selected with specificity and sensitivity preferably both ⩾0.70, favouring sensitivity.

#### Distribution-based methods

The SEM, 1.96SEM and 0.5s.d. of the change for each instrument were calculated. intra class coefficient values were obtained from the literature: 0.94 (CCQ), 0.80 (CAT) and 0.91 (SGRQ).^4–6^

#### Pooled MCID estimates

The mean estimates for the CCQ, CAT and SGRQ derived from patient-referencing, criterion-referencing, questionnaire-referencing, the SEM and 0.5s.d. were multiplied with a factor 1/5 each to calculate a pooled average. Domain scores were averaged based on patient- and criterion-referencing results.

## Figures and Tables

**Table 1:** Baseline characteristics

	*Baseline*
Age (years)^a^	57.87±6.56
BMI^a^	26.82±6.56
Gender (male)^b^	65.0 (*n*=293)
FEV_1_%pred^a^	50.40±15.11
GOLD II^b^	227 (50.3)
GOLD III^b^	176 (39.0)
GOLD IV^b^	48 (10.6)
Smoking pack-years^a^	42.61±23.47
Never smokers^b^	6 (1.3)
Active smokers^b^	179 (39.7)
Ex-smokers^b^	266 (59.0)
Retired^b^	74 (16.4)
If not retired, unable to work^b^	159 (35.3)
Patients with ⩾1 exacerbation(s) during the past 12 months before PR^b^	353 (78.4)

*N*=451.

Abbreviations: BMI, body mass index; FEV_1_%pred, forced expiratory volume in 1 s % predicted; GOLD, global initiative for chronic obstructive lung disease; PR, pulmonary rehabilitation.

a Data were expressed as mean±s.d.

b Data were expressed as frequencies (% of total patients).

**Table 2:** Health status outcomes of pulmonary rehabilitation

*Instrument*	*Baseline*^a^	*Change*^a^	*95% CI*^b^
*CCQ*			
Symptoms	2.87±1.24	−0.59±1.16	−0.70 to −0.48
Functional	2.86±1.34	−0.56±1.00	−0.65 to −0.46
Mental	2.86±1.74	−0.62±1.49	−0.76 to −0.48
Total	2.86±1.17	−0.58±0.92	−0.67 to −0.50
			
*CAT*			
Total	20.23±7.33	−3.11±5.59	−3.63 to −2.59
			
*SGRQ*			
Symptoms	63.66±21.77	−14.22±21.69	−16.24 to −12.21
Activities	63.58±19.82	−6.71±13.44	−7.96 to −5.47
Impact	39.21±18.81	−8.78±13.95	−10.08 to −7.49
Total	50.69±17.33	−9.04±12.11	−10.17 to −7.92
			
6MWD	427.73±110.18	80.19±54.72	75.01–85.37

*N*=451.

Abbreviations: CAT, COPD Assessment Test; CCQ, Clinical COPD Questionnaire; COPD, chronic obstructive pulmonary disease; mMRC, modified Medical Research Council Dyspnoea Scale; SGRQ, St George’s Respiratory Questionnaire; 6MWD, 6-min walking distance; 95% CI, 95% confidence interval;.

a Data were expressed as mean±s.d. Negative change represents improvement on the CCQ, CAT and SGRQ.

b Paired *t*-tests were applied for the normally distributed data, Wilcoxon signed-rank tests for non-parametric data. All tests were significant with *P*<0.05 comparing pre- and post-pulmonary rehabilitation scores.

**Table 3:** MCID patient-referencing results

*Instrument*	*No/hardly any improvement (GRC −1, 0 or +1) N*=*98*		*Minimal improvement (GRC +2 or +3) N*=*196*		*Moderate improvement (GRC +4 or +5) N*=*123*		*Major improvement (GRC +6 or +7) N*=*25*	
	*Δ*^a^	*95% CI*^b^	*Δ*^a^	*95% CI*^b^	*Δ*^a^	*95% CI*^b^	*Δ*^a^	*95% CI*^b^
*CCQ*								
Symptoms	−0.32	−0.54 to −0.10	−0.55	−0.70 to −0.40	−0.76	−0.96 to −0.56	−1.48	−1.97 to −0.99
Functional	−0.27	−0.47 to −0.07	−0.55	−0.68 to −0.43	−0.78	−0.98 to −0.58	−0.97	−1.31 to −0.63
Mental	−0.53	−0.84 to −0.22	−0.58	−0.78 to −0.38	−0.67	−0.94 to −0.39	−1.34	−1.84 to −0.84
Total	−0.34	−0.52 to −0.15	−0.56	−0.68 to −0.44	−0.75	−0.92 to −0.58	−1.25	−1.54 to −0.96
								
*CAT*								
Total	−2.05	−3.13 to −0.98	−3.12	−3.86 to −2.37	−3.67	−4.70 to −2.67	−6.44	−8.99 to −3.89
								
*SGRQ*								
Symptoms	−7.03	−10.86 to −3.19	−13.12	−16.05 to −10.19	−19.91	−23.92 to −15.90	−30.62	−38.40 to −22.84
Activities	−3.03	−5.28 to −0.78	−5.9	−7.88 to −4.08	−10.33	−12.78 to −7.87	−12.66	−18.69 to −6.62
Impact	−6.72	−9.57 to −3.86	−8.24	−10.14 to −6.33	−10.32	−12.77 to −7.87	−17.90	−23.64 to −12.16
Total	−5.57	−7.79 to −3.35	−8.40	−10.07 to −6.73	−11.83	−14.00 to −9.66	−18.50	−22.81 to −14.18

Abbreviations: CAT, COPD assessment test; CCQ, Clinical COPD Questionnaire; CI, confidence interval; GRC, global rating of change; MCID, minimal clinically important difference; SGRQ, St. George’s Respiratory Questionnaire; Δ, change score; 95% CI, 95% confidence interval.

a Data were reported as mean change scores. Negative change represents improvement for all instruments.

b Paired *t*-tests were applied to normally distributed variables, and Wilcoxon signed-rank tests were used for not normally distributed data. Data were reported as 95% CI. All change scores were significant with *P*-value <0.05.

**Table 4:** MCID questionnaire-referencing results

	*Anchor CCQ=0.40*	*Anchor CCQ=0.50*	*Anchor CAT=2*	*Anchor CAT=3*	*Anchor SGRQ=4*	*Anchor SGRQ=7*
*Regression analysis*						
CCQ	—	—	0.48 (0.42–0.53)	0.57 (0.53–0.61)	0.34 (0.28–0.40)	0.48 (0.44–0.53)
CAT	2.45 (2.14–2.77)	2.81 (2.54–3.08)	—	—	1.86 (1.46–2.27)	2.61 (2.32–2.91)
SGRQ	7.51 (6.86–8.16)	8.35 (7.79–8.90)	7.73 (6.98–8.48)	8.89 (8.31–9.47)	—	—
						
*Failing/achieving*						
CCQ	—	—	0.48	0.56	0.46	0.53
CAT	2.74	2.82	—	—	2.45	2.86
SGRQ	8.14	8.36	7.78	8.69	—	—
						
*ROC curves*						
CCQ	—	—	0.50	0.60	0.50	0.60
			AUC 0.767	AUC 0.771	AUC 0.796	AUC 0.802
			Sens 0.701	Sens 0.716	Sens 0.750	Sens 0.763
			Spec 0.706	Spec 0.710	Spec 0.714	Spec 0.730
CAT	3.00	3.00	—	—	3.00	3.00
	AUC 0.768	AUC 0.770			AUC 0.722	AUC 0.737
	Sens 0.726	Sens 0.729			Sens 0.727	Sens 0.705
	Spec 0.656	Spec 0.668			Spec 0.605	Spec 0.650
SGRQ	8.30	8.63	7.50	8.00	—	—
	AUC 0.817	AUC 0.816	AUC 0.719	AUC 0.745		
	Sens 0.777	Sens 0.787	Sens 0.659	Sens 0.673		
	Spec 0.703	Spec 0.702	Spec 0.656	Spec 0.681		

Data are expressed as estimates (95% CI).

*N*=451;

Abbreviations: AUC, area under the curve; CAT, COPD Assessment Test; CCQ, Clinical COPD Questionnaire; COPD, chronic obstructive pulmonary disease; ROC, receiver operating characteristics curves; Sens, Sensitivity; Spec, Specificity; SGRQ, St. George’s Respiratory Questionnaire; 95% CI, 95% confidence interval.
