# Supplementary material for: Health status instruments for patients with COPD in pulmonary rehabilitation: defining a minimal clinically important difference
Source: NPJ Prim Care Respir Med. 2016 Sep 1;26:16041–. doi: 10.1038/npjpcrm.2016.41 (PMC5011855; doi:10.1038/npjpcrm.2016.41)
Supplement: Supplementary Information [file npjpcrm201641-s1.doc]

# Online supplement

| **Table 1: Relevant health status correlations** | | | | | | | |  | |
| --- | --- | --- | --- | --- | --- | --- | --- | --- | --- |
| **Instrument** |  | | | | | |  | | |
|  | **CAT** | **SGRQ** | |  | |  |  | | |
| **CCQ** |  | Symptoms | Activity | | Impact | | Total | |  |
| Symptoms | 0.47 | 0.51* | 0.33 | | 0.38 | | 0.50* | | |
| Functional | 0.45 | 0.39 | 0.43 | | 0.47 | | 0.55* | | |
| Mental | 0.36 | 0.27 | 0.29 | | 0.43 | | 0.43 | | |
| Total | 0.59* | 0.50* | 0.44 | | 0.54* | | 0.63* | | |
|  |  |  |  | |  | |  | | |
| **CAT** | - | 0.40 | 0.33 | | 0.41 | | 0.54* | | |
| N= 451  Data expressed as correlation coefficients. All correlations significant at p<0.05.  * Correlations ≥0.50.  CAT, COPD Assessment Test; CCQ, Clinical COPD Questionnaire; SGRQ, St. George’s Respiratory Questionnaire | | | | | | | |  | |

| **Table 2: Summary MCID results** | | | |
| --- | --- | --- | --- |
|  | **CCQ** | **CAT** | **SGRQ** |
| **Estimate from literature** | 0.40 | 2.00 | 4.00 |
|  |  |  |  |
| **Patient-referencing** | 0.56  *0.55 symptoms*  *0.55 functional*  *0.58 mental* | 3.12 | 8.40  *13.12 symptoms*  *5.98 activity*  *8.24 impact* |
| **Criterion-referencing** | 0.62  *0.47 symptoms*  *0.67 functional*  *0.86 mental* | 2.96 | 9.28  *- symptoms*  *10.61 activity*  *9.93 impact* |
|  |  |  |  |
| **Questionnaire-referencing ranges** |  |  |  |
| **SGRQ=4 and SGRQ=7 as anchor** |  |  |  |
| Scatter plots / regression | 0.28-0.53 | 1.46-2.91 |  |
| Mean failing/achieving estimate | 0.46-0.53 | 2.45-2.86 |  |
| ROC Curves | 0.50-0.60 | 3.00-3.00 |  |
|  |  |  |  |
| **CAT=2 and CAT=3 as anchor** |  |  |  |
| Scatter plots / regression | 0.42-0.61 |  | 6.98-9.47 |
| Mean failing/achieving estimate | 0.48-0.56 |  | 7.78-8.69 |
| ROC Curves | 0.50-0.60 |  | 7.50-8.00 |
|  |  |  |  |
| **CCQ=0.40 and CCQ=0.50 as anchor** |  |  |  |
| Scatter plots / regression |  | 2.14-3.08 | 6.86-8.90 |
| Mean failing/achieving estimate |  | 2.74-2.82 | 8.14-8.36 |
| ROC Curves |  | 3.00-3.00 | 8.30-8.63 |
|  |  |  |  |
| **Distribution-based approaches** |  |  |  |
| SEM | 0.29 | 3.28 | 5.20 |
| 1.96SEM | 0.56 | 6.43 | 10.19 |
| 0.5SD | 0.46 | 2.80 | 6.06 |
|  |  |  |  |
| **Pooled estimate** | 0.52  *0.51 symptoms*  *0.61 functional*  *0.72 mental* | 3.29 | 7.91  *13.12 symptoms*  *8.30 activity*  *9.09 impact* |
|  |  |  |  |
| N=451  0.5SD, Half Standard Deviation; CAT, COPD Assessment Test; CCQ, Clinical COPD Questionnaire; MCID, Minimal Clinically Important Difference; ROC, Receiver Operating Characteristics Curves; SEM, Standard Error of Measurement; SGRQ, St. George’s Respiratory Questionnaire | | | |

**SCATTER PLOTS and REGRESSION ANALYSIS**

**Legenda Scatter Plots:**

- Red lines are MCID estimates from the literature (SGRQ -4.00, CCQ -0.40, CAT -2.00).
- Green lines are MCID estimate based upon the rounded average from patient-referencing, criterion-referencing and the distribution-based methods (SGRQ -7.00, CCQ -0.50, CAT -3.00)

**MCID of the SGRQ**

*Figure 1: SGRQ with CCQ as anchor*


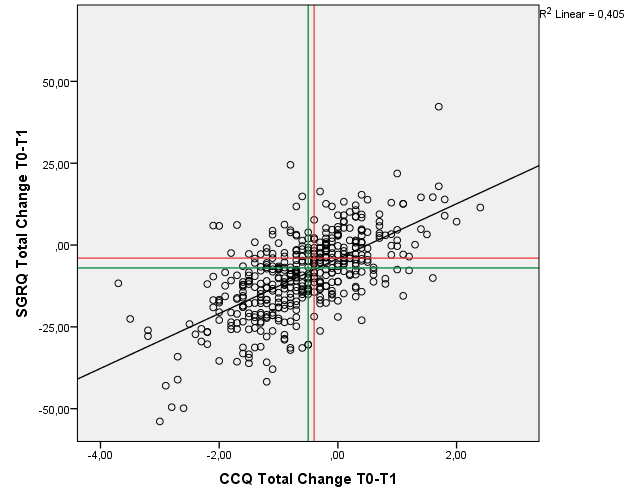


**Base formula: SGRQ Total Change Score = -4.153 + 8.384 * CCQ total change score**

**95% CI formulas:**

**Lower bound SGRQ Total Change Score = -5.182 + 7.438 * CCQ total change score**

**Upper bound SGRQ Total Change Score = -3.123 + 9.330 * CCQ total change score**

**MCID of the SGRQ**

*Figure 2: SGRQ with CAT as anchor*


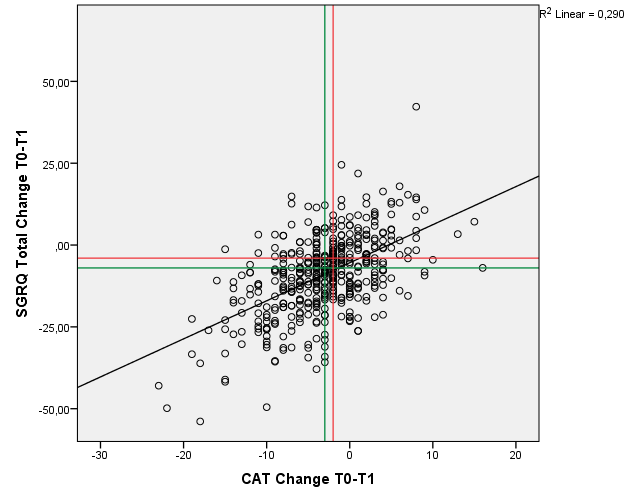


**Base formula SGRQ Total Change Score = -5.404 + 1.163 * CAT total change score**

**95% CI formula**

**Lower bound SGRQ Total Change Score = -6.492 + 0.993 * CAT total change score**

**Upper bound SGRQ Total Change Score = -4.315 + 1.333 * CAT total change score**

**MCID of the CCQ**

*Figure 3: CCQ with SGRQ as anchor*


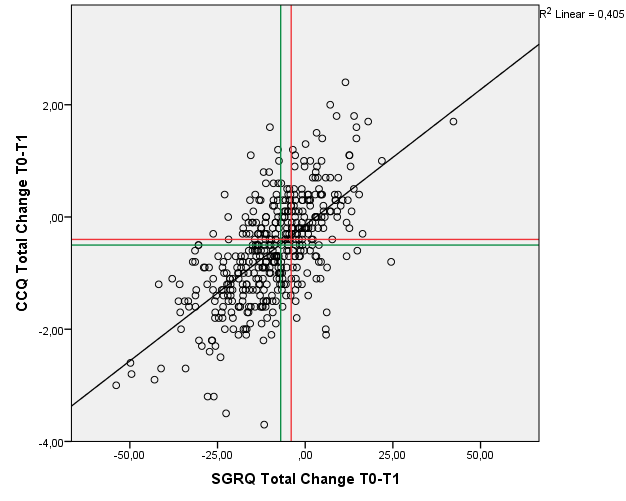


**Base formula CCQ Total Change Score = -0.146 + 0.048 * SGRQ total change score**

**95% CI formula**

**Lower bound CCQ Total Change Score = -0.228 + 0.043 * SGRQ total change score**

**Upper bound CCQ Total Change Score = -0.064 + 0.054 * SGRQ total change score**

**MCID of the CCQ**

*Figure 4: CCQ with CAT as anchor*


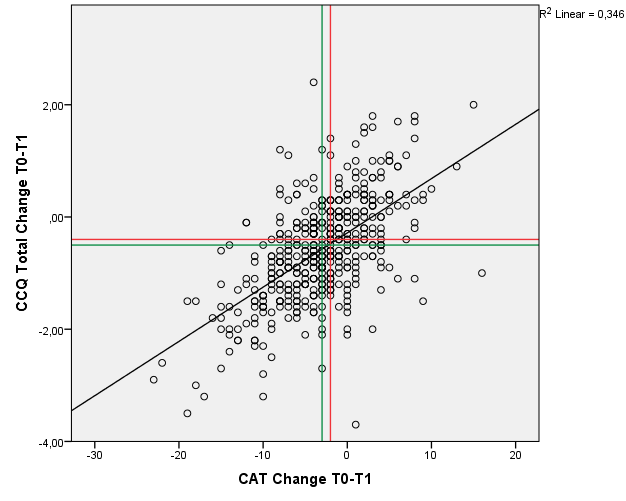


**Base formula CCQ Total Change Score = -0.283 + 0.097 * CAT total change score**

**95% CI formula**

**Lower bound CCQ Total Change Score = -0.362 + 0.084 * CAT total change score**

**Upper bound CCQ Total Change Score = -0.204 + 0.109 * CAT total change score**

**MCID of the CAT**

*Figure 5: CAT with SGRQ as anchor*


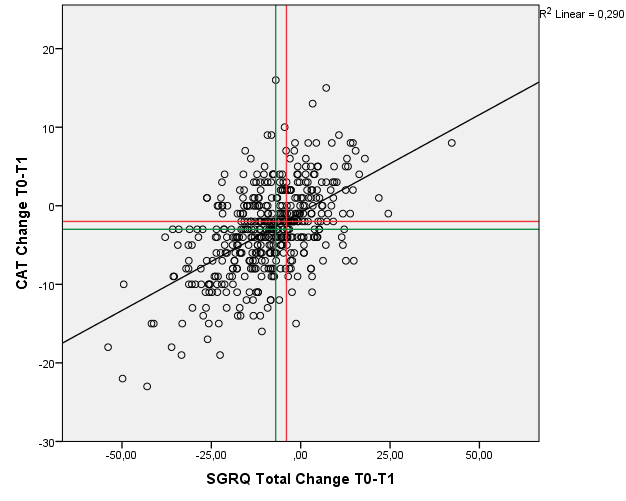


**Base formula CAT Total Change Score = -0.865 + 0.249 * SGRQ total change score**

**95% CI formula**

**Lower bound CAT Total Change Score = -1.415 + 0.213 * SGRQ total change score**

**Upper bound CAT Total Change Score = -0.316 + 0.286 * SGRQ total change score**

**MCID of the CAT**

*Figure 6: CAT with CCQ as anchor*


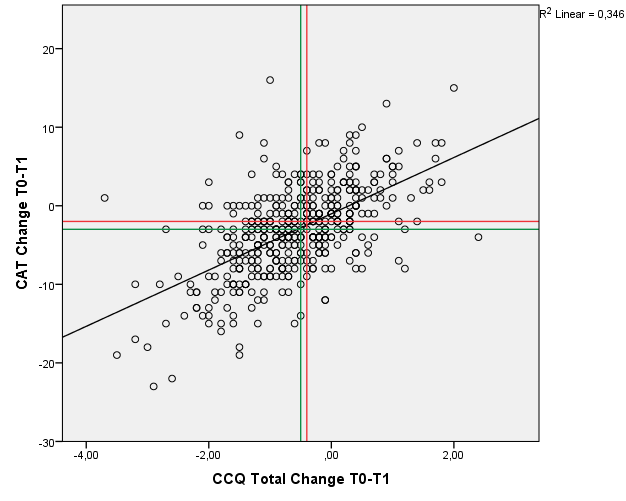


**Base formula CAT Total Change Score = -1.020 + 3.581 * CCQ total change score**

**95% CI formula**

**Lower bound CAT Total Change Score = -1.518 + 3.124 * CCQ total change score**

**Upper bound CAT Total Change Score = -0.523 + 4.039 * CCQ total change score**
